# Methyl-β-Cyclodextrin Impairs the Phosphorylation of the β_2_ Subunit of L-Type Calcium Channels and Cytosolic Calcium Homeostasis in Mature Cerebellar Granule Neurons

**DOI:** 10.3390/ijms19113667

**Published:** 2018-11-20

**Authors:** Sofia Fortalezas, Dorinda Marques-da-Silva, Carlos Gutierrez-Merino

**Affiliations:** Department of Biochemistry and Molecular Biology of the Faculty of Sciences and Institute of Molecular Pathology Biomarkers, University of Extremadura, 06006-Badajoz, Spain; sofia_furta@hotmail.com (S.F.); dorindams@gmail.com (D.M.-d.-S.)

**Keywords:** β_2_ subunit of L-type calcium channels, cholesterol depletion, caveolin-1-rich lipid rafts, PKA, CaMK-II, cytosolic calcium homeostasis, cerebellar granule neurons

## Abstract

The activation of L-type calcium channels (LTCCs) prevents cerebellar granule neurons (CGNs) from entering low-K^+^-induced apoptosis. In previous works, we showed that LTCCs are largely associated with caveolin-1-rich lipid rafts in the CGN plasma membrane. In this work, we show that protein kinase A (PKA) and calmodulin-dependent protein kinase II (CaMK-II) are associated with caveolin-1-rich lipid rafts of mature CGNs, and we further show that treatment with the cholesterol-trapping and lipid raft-disrupting agent methyl-β-cyclodextrin decreases the phosphorylation level of the LTCC β_2_ subunit and the steady-state calcium concentration in neuronal somas ([Ca^2+^]_i_) to values close to those measured in 5 mM KCl proapoptotic conditions. These effects correlate with the effects produced by a short (15 min) treatment of CGNs with H-89 and KN-93—inhibitors of PKA and CaMK-II, respectively—in 25 mM KCl medium. Moreover, only a 15 min incubation of CGNs with H-89 produces about a 90% inhibition of the calcium entry that would normally occur through LTCCs to increase [Ca^2+^]_i_ upon raising the extracellular K^+^ from 5 to 25 mM, i.e., from proapoptotic to survival conditions. In conclusion, the results of this work suggest that caveolin-1-rich lipid rafts play a major role in the control of the PKA- and CaMK-II-induced phosphorylation level of the LTCC β_2_ subunit, thus preventing CGNs from entering apoptosis.

## 1. Introduction

Cyclodextrins are used as additives to improve the aqueous solubility of poorly soluble drugs and increase their bioavailability [1], and they are being increasingly used in nanoparticle-based drug delivery [2]. Cyclodextrins are also used as anti-browning agents in different foods and foodstuffs [3]. However, a link between cyclodextrins and iatrogenic hearing loss has been noted in several species, including humans [4]. In particular, methyl-β-cyclodextrin has been widely used for the selective removal of cholesterol and disruption of lipid rafts in different cell lines (see, e.g., [5,6]). The neurological risks of using methyl-β-cyclodextrin are informed by the reported toxicity of this compound to neuronal cell lines [7]. Thus, the molecular mechanism underlying selective neuronal cell death induced by methyl-β-cyclodextrin deserves to be studied. 

L-type calcium channels (LTCCs) together with *N*-methyl d-aspartate receptors (NMDAr), plasma membrane Ca^2+^-ATPase (PMCA), and sodium calcium exchangers are associated with the cholesterol and caveolin-1-rich lipid raft nanodomains of the cerebellar granule neuron (CGN) plasma membrane [8,9,10], thereby inducing high calcium transients near these nano-transducer domains that potentiate faster and stronger Ca^2+^-mediated neuronal responses to external stimulus. The pore-forming α_1_ subunits (from Ca_V_1.1 to Ca_V_1.4) determine the electrophysiological and pharmacological properties of LTCCs, but auxiliary subunits (β_1–4_, α_2_-δ and γ) modulate their trafficking, surface expression, and biophysical properties [11,12,13]. β subunits have been shown to serve as scaffolding proteins that bind AHNAKs to link voltage-gated calcium channels (VGCCs) to the actin cytoskeleton [14]. In a previous work, we showed that disruption of the actin cytoskeleton in cultured CGNs exposed to 3-morpholinosydnonimine-oxidative stress is linked to alterations of the cytosolic calcium concentration [15]. Notably, both protein kinase A (PKA) and calcium/calmodulin-dependent protein kinase II (CaMK-II) are also associated with cytoskeleton elements near the plasma membrane. Razani and colleagues demonstrated the colocalization and direct interaction between the caveolin-1 scaffolding domain and the catalytic subunit of PKA in vitro and in vivo [16]. Further, in the brain, the association of PKA with the LTCC Ca_V_1.2 (α_1C_) subunit was demonstrated [17]. Several works have reported that CaMK-II interacts with LTCCs and NMDAr [18,19,20]. Moreover, it has been suggested that a direct association exists between CaMK-II and lipid rafts [21], as well as a colocalization between LTCC (Ca_V_1.2) and CaMK-II [22]. LTCC modulation by PKA is due to β_2_ and Ca_V_1.2 (α_1C_) subunit phosphorylation [23,24]. Mutations of serine 478 (Ser478) and serine 479 (Ser479) in the β_2_ subunit were shown to completely inhibit the increase in calcium currents through LTCCs induced by PKA [23]. LTCC modulation by CaMK-II plays a major role in the calcium-dependent facilitation of these calcium channels [25,26]. Moreover, the association of CaMK-II with the LTCC β subunit facilitates the interaction of CaMK-II with additional domains of the α_1_ subunit [25] and can enhance phosphorylation by CaMK-II at Thr498 and other residues of the β_2a_ subunit [27]. Of significant note are reports that the deregulation of lipid raft-dependent signaling is likely to play a major role in neurodegenerative diseases characterized by alterations of cholesterol and ganglioside composition, such as Alzheimer’s, Parkinson’s, and other age-related diseases [28,29,30,31].

The control of cytosolic calcium is a key factor for cell survival [32]. In primary cultures of mature cerebellar granule neurons (CGNs), the entry of calcium through L-type calcium channels (LTCCs) plays a major role in maintaining cytosolic calcium homeostasis within the survival concentration range [33,34,35]. Lowering the extracellular potassium concentration to 5 mM leads to a slowly developing apoptotic process in CGNs cultured with a sustained low level of [Ca^2+^]_i_ [33]. Activation of voltage-operated Ca^2+^ channels has been shown to account for most of the increase in [Ca^2+^]_i_ observed in CGNs upon partial depolarization by increasing KCl concentration to 25 mM [33,36,37,38]; this dependency also explains the proapoptotic effects of blocking LTCCs by nifedipine or nimodipine [33]. In addition, PKA and CaMK have been reported to play a major role in the protection against low-potassium-induced CGN apoptosis in vitro. Cyclic AMP is protective against apoptosis: when apoptosis is induced by shifting cultures to low potassium, cAMP activates cAMP-dependent PKA [39,40,41,42]. CaMK-mediated activation of the protein kinase B pathway promotes CGN survival [43], and it has been shown that calcium entry through LTTCs, which protects cells from low-potassium-induced apoptosis, potentiates the activity of calcium/calmodulin kinase IV (CaMK-IV), preventing caspase 3-dependent cleavage of CaMK-IV, which in turn maintains a survival–favoring level of CREB-dependent gene expression [44]. However, in previous works, the possibility that the protective effects afforded by PKA and CaMK activities were, at least in part, accounted for by phosphorylation-dependent modulation of LTCCs has not been considered, to the best of our knowledge, and deserves to be studied. 

On these grounds, the major aims of this work are to study in mature CGNs cultured on a chemically defined medium: (i) the effect of the lipid raft-disrupting agent methyl-β-cyclodextrin (MβCD) on the phosphorylation level of the β_2_ subunit of LTTCs and on the steady-state cytosolic calcium concentration; (ii) the phosphorylation level of the β_2_ subunit of LTTCs in 25 mM KCl medium, in proapoptotic 5 mM KCl medium, and in the presence of PKA and CaMK-II inhibitors; and (iii) the effect of inhibitors of PKA and CaMK-II on the steady-state cytosolic calcium concentration.

## 2. Results

### 2.1. Treatment of CGNs with MβCD Decreases the Phosphorylation Level of the β_2_ Subunit of LTCC

The experimental conditions for significant cholesterol sequestration by MβCD were fixed in this work while taking into account the average duration of the CGN treatments with protein kinase inhibitors (15 min) plus the time for the acquisition of 340/380 ratio images (5 min). Thus, we incubated the CGNs with different concentrations of MβCD at 37 °C for 20 min, followed by removal of the medium to analyze the cholesterol content of neurons. The results shown in Table 1 demonstrate that a 20 min exposure of CGNs to 10 mM MβCD at 37 °C is enough to reduce their cholesterol content from 22.8 ± 2.1 to 4.6 ± 0.4 nmol cholesterol/mg of CGN protein, i.e., more than an 80% reduction in the cholesterol content of CGNs. These results also point out that the maximum sequestration of cholesterol from these cells is obtained with 10 mM MβCD under experimental conditions that do not produce a significant loss of cell viability after 20 min (Table 1). Indeed, concentrations of MβCD higher than 20 mM were needed to observe a significant loss of cell viability within this time period. These experimental conditions are similar to those used in a previous work, where we showed that a large impairment of the NMDAr response to l-glutamate is induced by MβCD treatment in mature CGNs [9].

**Figure 1:**
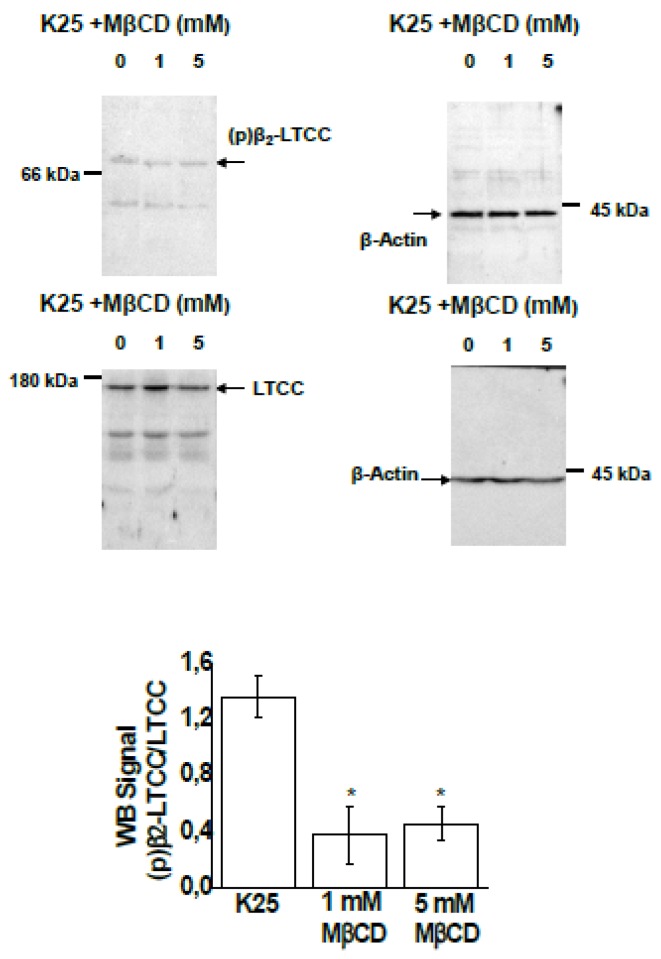
MβCD decreases the phosphorylation level of the β_2_ subunit of L-type calcium channels (LTCCs). The phosphorylation level of the β_2_ subunit of LTCCs was measured by Western blotting, as indicated below, after the incubation of CGNs in MLocke’s K25 plus 1 or 5 mM of MβCD for 15 min at 37 °C in a 5% CO_2_ culture chamber. After SDS-PAGE (7.5% acrylamide), the proteins were transferred to PVDF membranes for Western blotting, as indicated in the Materials and Methods. The positions of the molecular weight marker bands closest to the target proteins of the primary antibodies, i.e., β_2_ subunit of LTCCs, LTCC α_1C_ subunit, and β-actin, are shown at the left or right side of the corresponding PVDF membrane images. The phosphorylation levels of the β_2_ subunit of LTCCs, (p)β_2_LTCC/LTCC, were measured using the ratio between the intensities of the ~73 and ~174 kDa bands obtained with rabbit antibodies: PCCb2-140AP (FabGennix Inc., Frisco, TX, USA)—dilution 1:100 [(p)β_2_LTCC]—and anti-LTCC-α_1C_-subunit (Santa Cruz Biotechnology, Heidelberg, Germany, sc-25686)—dilution 1:100 [LTCC], respectively. For quantification of the ratio (p)β_2_LTCC/LTCC, the intensities of the bands were normalized first to the intensities of their corresponding ~42 kDa anti-β-actin bands (mouse anti-β-actin A1978, 0.75 µg/mL) as the internal control of protein loading. See Materials and Methods for other experimental details. Images shown are representative of the results obtained in experiments done with at least three different CGN preparations. The average results ± S.E. of triplicate experiments are presented as a bar plot. (*) *p* < 0.05.

Treatment with MβCD largely alters the phosphorylation levels of LTCCs in CGNs (Figure 1). Compared to the control conditions (K25), treatment with 1 and 5 mM MβCD decreases the phosphorylation level of the β_2_ subunit of LTCCs by 60–70%.

### 2.2. PKA and CaMK-II Are Associated with Caveolin-1-Rich Lipid Rafts in the Plasma Membrane of CGNs

To evaluate the relative distribution of proteins between lipid raft and non-lipid raft membrane fractions, we experimentally assessed: (1) the presence of PKA and CaMK-II in caveolin-1-rich lipid rafts prepared from of mature CGNs in culture and (2) the percentage of immunoprecipitation of PKA and CaMK-II with caveolin-1 in cell lysates treated with 1% Triton X-100.

**Figure 2:**
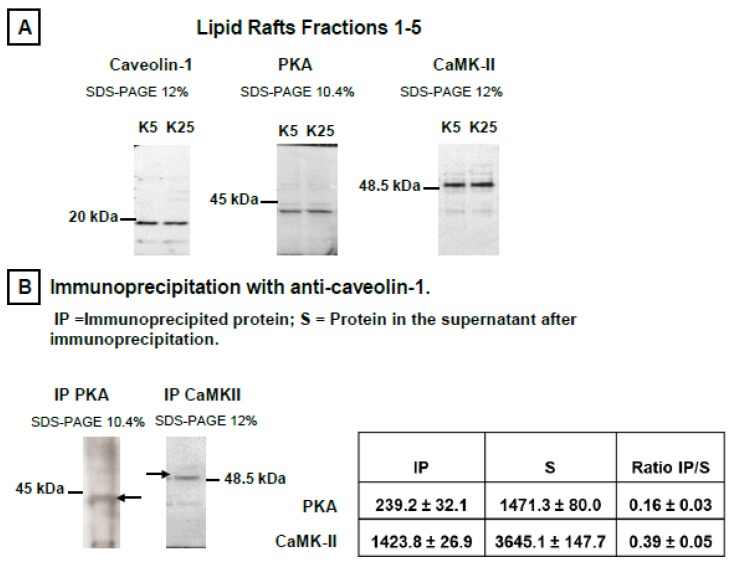
Protein kinase A (PKA) and calmodulin-dependent protein kinase II (CaMK-II) are associated with lipid rafts isolated from mature CGNs in culture. The presence of PKA and CaMK-II in lipid raft membrane fractions was determined by Western blotting using rabbit anti-PKA (sc-28892) and rabbit anti-CaMK-II (sc-9035), with rabbit anti-caveolin-1 (sc-894) for the lipid raft protein marker caveolin-1. Fractions 1–5, which are enriched in lipid raft marker proteins caveolin-1 and H-Ras, are also enriched in cholesterol, as shown in previous works by our laboratory [8,9,10]. (**A**) PKA and CaMK-II are present in fractions 1–5 where lipid raft markers are also enriched, both in CGN survival medium MLocke’s K25 and also after 1 h incubation at 37 °C in a 5% CO_2_ culture chamber in proapoptotic MLocke’s K5 medium. The Western blot images shown are representative of the results obtained in experiments done with at least three different preparations of lipid rafts, as indicated in Materials and Methods. The same amount of lipid raft protein, 5 μg, was loaded into each lane in these experiments. The position of the molecular weight marker bands closest to the target proteins of the primary antibodies, i.e., caveolin-1 (~20 kDa), CaMK-II (~50 kDa), and PKA (~40 kDa), are shown at the left or right side of the corresponding PVDF membrane images. (**B**) Results of immunoprecipitation of cell lysates with caveolin-1, performed as indicated in Materials and Methods. The fraction of PKA and CaMK-II immunoprecipitated with caveolin-1 was calculated from a densitometry analysis of their bands in the Western blots and revealed using the primary and secondary antibodies indicated above, taking into account that while all precipitated samples were loaded into the corresponding lanes of the SDS-PAGE gels (IP), only a fraction of the total supernatant volume (S) was loaded into the gel lanes. The results shown in the table inserted in this panel are the average ± S.E. of triplicate experiments.

Lipid rafts were prepared as indicated in the Materials and Methods section from mature CGNs in MLocke’s K5 (proapoptotic conditions) and also in partially depolarizing plasma membrane media, K25 (survival conditions). Lipid raft fractions were characterized using H-Ras, caveolin-1, and flotillin as protein markers, as in previous works [8,9,10]. Western blots of lipid raft fractions show that PKA and CaMK-II are present in lipid rafts prepared from CGNs in MLocke’s K5 and K25 (Figure 2A). Note, however, that these protein kinases are not only present in the lipid raft fractions, since PKA and CaMK-II are also present in other membrane fractions.

Immunoprecipitation of cell lysates with caveolin-1 was carried out as indicated in the Materials and Methods section, and the results are presented in image and table format in Figure 2B. The results show that both PKA and CaMK-II coimmunoprecipitate with anti-caveolin-1. The intensity of Western blot bands was calculated for the whole immunoprecipitate and supernatant fractions to calculate the percentage of PKA and CaMK-II present in the total cell lysate that is bound to caveolin-1 or to proteins associated with caveolin-1. These results also highlight that CaMK-II has a higher percentage of coimmunoprecipitation with caveolin-1 (39 ± 5%) than PKA (16 ± 2%).

### 2.3. PKA and CAMK-II Inhibitors Decrease the Phosphorylation Level of the β_2_ Subunit of LTCCs to the Phosphorylation Levels Measured after CGN Treatment with MβCD and Also in Proapoptotic K5 Medium

**Figure 3:**
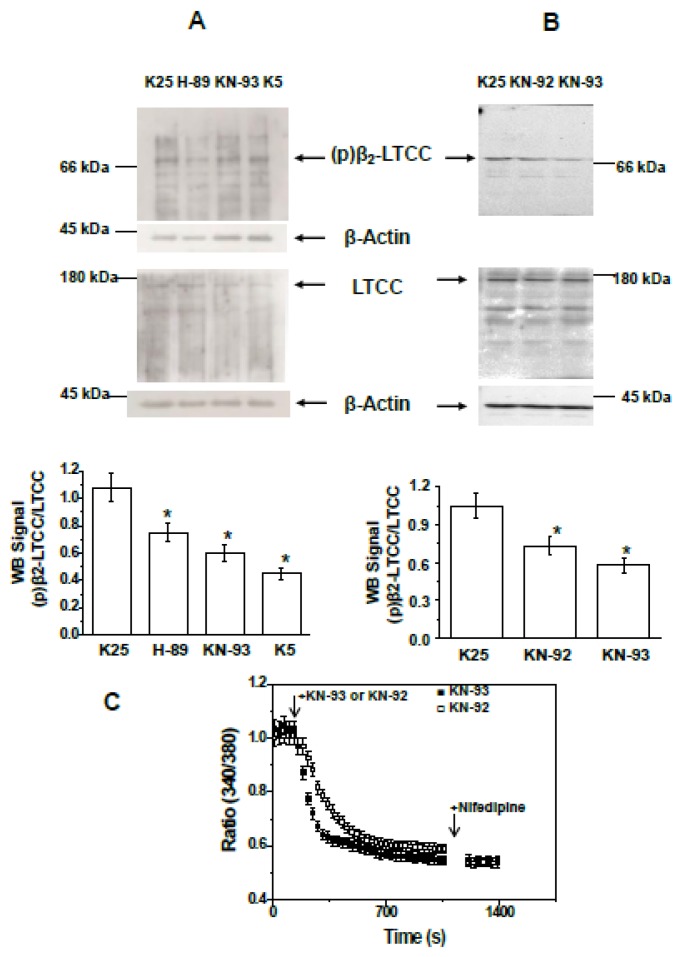
H-89 and KN-93 decrease the phosphorylation of the β_2_ subunit of LTCCs to values measured in proapoptotic MLocke’s K5. (**A**) CGNs in MLocke’s K25 were incubated for 15 min at 37 °C in a 5% CO_2_ culture chamber with 20 µM H-89, 30 µM KN-93, or in the absence of these inhibitors (K25 control), or after CGN incubation for 1 h at 37 °C in the 5% CO_2_ culture chamber in the proapoptosis MLocke’s K5 condition (K5). (**B**) CGNs in MLocke’s K25 were incubated for 15 min at 37 °C in the 5% CO_2_ culture chamber with 30 µM KN-92, 30 µM KN-93, or in the absence of these inhibitors (K25 control). After SDS-PAGE (7.5% acrylamide), the proteins were transferred to PVDF membranes for Western blotting as indicated in Materials and Methods. For panel B, the PVDF membrane was stripped twice to remove primary antibodies—anti-β_2_ subunit of LTCCs and anti-LTCC α_1C_ subunit—and used afterward for β-actin detection. The results shown in these panels are representative of the results obtained in triplicate experiments with different CGN preparations. The position of the molecular weight marker bands closest to the target proteins of the primary antibodies, i.e., β_2_ subunit of LTCC, LTCC α_1C_ subunit, and β-actin, are shown at the left or right side of the corresponding PVDF membrane images. Western blots were revealed by exposure to autoradiography films for (**A**) and with ChemiDoc^TM^ XRS+ from Bio-Rad for (**B**). The phosphorylation levels of the β_2_ subunit of LTCCs, (p)β_2_LTCC/LTCC, shown in the bar graphs of panels A and B, were measured as indicated in the legend for Figure 1 and are average results ± S.E. of triplicate experiments. (*) *p* < 0.05. (**C**) Time dependence of population averages of the 340/380 ratio in neuronal somas before and after the addition of 30 µM KN-93 (black-filled squares) or 30 µM KN-92 (white-filled squares) at the point indicated by the first arrow. At the point indicated by the second arrow, 10 µM nifedipine was added. Mature CGNs in culture were loaded with Fura-2, as indicated in the Materials and Methods section, and then changed to MLocke’s K25 buffer (37 °C) to start serial 340 and 380 image acquisition. Data acquisition was done as indicated in the Materials and Methods section, with exposure times lower than 0.4 s at time intervals of 30 s. The 340/380 ratio of neuronal somas was measured using the region of interest (ROI) tool of the HCImage software. The 340/380 ratio values shown are the average ± S.E. of experiments done with three different preparations of CGNs (n > 300 neuronal somas of fields taken from six plates for each experimental condition).

The effects of pre-incubating CGNs with the PKA inhibitor H-89, the CaMKII inhibitor KN-93, and the KN-93 analogue KN-92 (noninhibitor) for 15 min on the steady-state level of the phosphorylation of the LTCC β_2_ subunit were measured by Western blotting using the antibody PCCb2-140AP (FabGennix Inc.). The results show that the phosphorylation level of the β_2_ subunit of LTCCs of mature CGNs in MLocke’s K25 is high, suggesting that LTCCs are largely phosphorylated in these experimental conditions (Figure 3A). The results in Figure 3A also show that the ratio (p)LTCC/LTCC decreases 30 ± 5% when CGNs are treated with the PKA inhibitor H-89 and 45 ± 5% when CGNs are treated with the CaMK-II inhibitor KN-93. Notably, the analogue of KN-93, KN-92, though not an inhibitor of CaMK-II, affords a decrease of 30 ± 3% in the (p)LTCC/LTCC ratio, which is about 2/3 of the decrease produced by KN-93 (Figure 3B). Taking into account the major role of LTCCs in controlling the steady-state concentration of cytosolic calcium in CGNs [34,35,45] and that both KN-92 and KN-93 are potent inhibitors of calcium entry through LTCCs [46], we hypothesized that at the concentrations of KN-93 and KN-92 used, these compounds can produce a large drop in CGN cytosolic calcium concentration, and this would elicit CaMK-II inhibition. This was confirmed by measuring the cytosolic calcium concentration of Fura-2-loaded CGNs after the addition of KN-92 and KN-93 (Figure 3C). The results show that (a) both produce a large decrease in the (340/380) ratio, from 1.0 ± 0.1 to 0.55 ± 0.05 for KN-93 and to 0.65 ± 0.05 for KN-92, values that are close to those attained after the addition of the LTCC blocker nifedipine, 0.55 ± 0.05, and (b) there is a slower rate [Ca^2+^]_i_ decrease in CGN somas for KN-92 than for KN-93. It is worthy to note that low (340/380) ratio values (close to 0.5) are also attained upon lowering the concentration of K^+^ in the extracellular medium to 5 mM [34,47]. Proapoptotic conditioning events develop as early as 1 h after changing these neurons to a K5 medium [45,47,48,49]. Notably, the phosphorylation level of the β_2_ subunit of LTCCs after 1 h of proapoptotic conditioning in MLocke’s K5 decreases by 65 ± 5% (Figure 3A). This decrease is higher than that observed after incubation of CGNs in MLocke’s K25 for 15 min with the CaMK-II inhibitor KN-93. However, the sum of the contributions of CaMK-II and PKA to the steady phosphorylation level in MLocke’s K25 is 80 ± 10% (see above), a value that can fully account for the decrease in phosphorylation of the β_2_ subunit of LTCCs observed after CGN treatment with MβCD or after 1 h in MLocke’s K5.

### 2.4. Treatment of CGNs with MβCD or with the PKA Inhibitor H-89 Lowers Steady-State [Ca^2+^]_i_ to Values Close to Those Attained in Proapoptotic K5 Medium

**Figure 4:**
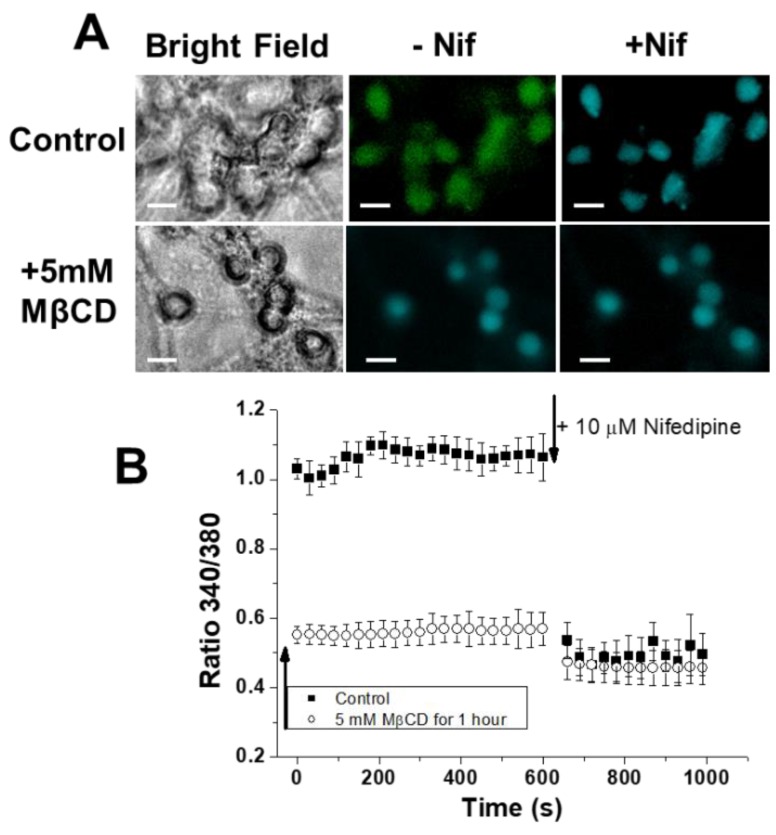
CGN treatment with MβCD decreases the steady-state [Ca^2+^]_i_ to values close to those measured after the addition of nifedipine. (**A**) Pre-incubation and removal of MβCD–cholesterol complexes decreases the 340/380 ratio of CGNs in MLocke’s K25 to values close to those measured after blocking LTCCs by nifedipine. Representative images of mature CGNs in culture (controls) and treated with 5 mM MβCD for 1 h at 37 °C in a 5% CO_2_ culture chamber in the maturation medium DMEM/F12 followed by a change of the DMEM/F12 medium to efficiently remove MβCD–cholesterol complexes (+5 mM MβCD). Thereafter, CGNs were loaded with Fura-2, as indicated in the Materials and Methods section, and then changed to MLocke’s K25 buffer at 37 °C, and 340/380 ratio images were captured at different times before (-Nif) and 1 min after the addition of 10 µM nifedipine (+Nif). Pseudocolor scale for 340/380 ratio values: light blue, 0.5–0.6; green, 1.0–1.1. Scale bar = 10 µm. (**B**) Time dependence of population averages of the 340/380 ratio in neuronal somas before and after the addition of 10 µM nifedipine at the time indicated by an arrow for CGNs treated with 5 mM MβCD in Panel A (open circles) and for controls (untreated CGN, solid black squares). The sequential 340/380 ratio images acquired at different times after the addition of nifedipine show that a new steady-state [Ca^2+^]_i_ is already reached at 1 min. Data acquisition and analysis were done after the selection of neuronal somas using the region of interest (ROI) tool of the HCImage software, as indicated in the Materials and Methods section, with exposure times lower than 0.4 s at time intervals of 30 s. The 340/380 ratio values are the average ± S.E. of experiments done with three different preparations of CGNs (n > 300 neuronal somas of fields taken from six plates for each experimental condition).

CGNs were treated with 5 mM MβCD for 1 h in serum-free DMEM:F12 medium (1:1), supplemented as indicated in Materials and Methods, to experimentally assess the effect of CGN treatment with MβCD on calcium entry through LTCCs. Then, the medium was replaced to remove MβCD–cholesterol complexes, and CGNs were loaded with 5 µM Fura-2 AM. As we noticed that this treatment with MβCD enhances the Fura-2 AM loading of CGNs, we used shorter times for loading in these experiments, between 30 and 35 min. After CGN loading with Fura-2 AM, we measured the cell viability as indicated in Materials and Methods and found that this treatment produces less than a 10% loss of cell viability, i.e., a statistically nonsignificant loss of cell viability. The 340/380 ratio measurements show that the treatment with MβCD lowers the steady-state [Ca^2+^]_i_ to values approaching those obtained after the addition of nifedipine (Figure 4A,B).

**Figure 5:**
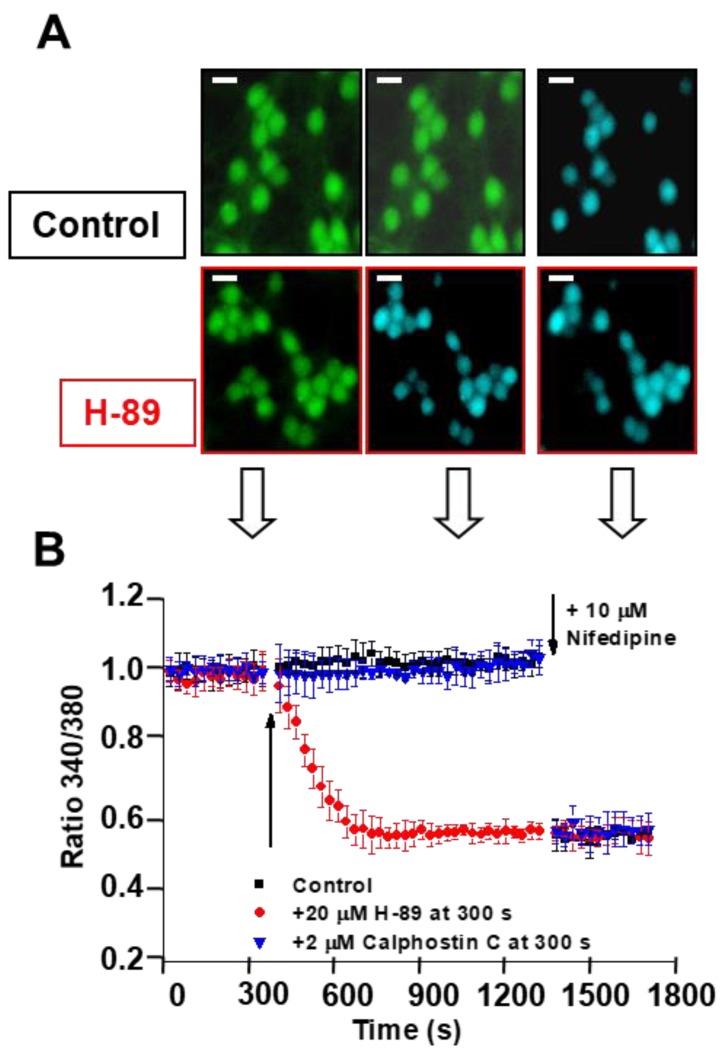

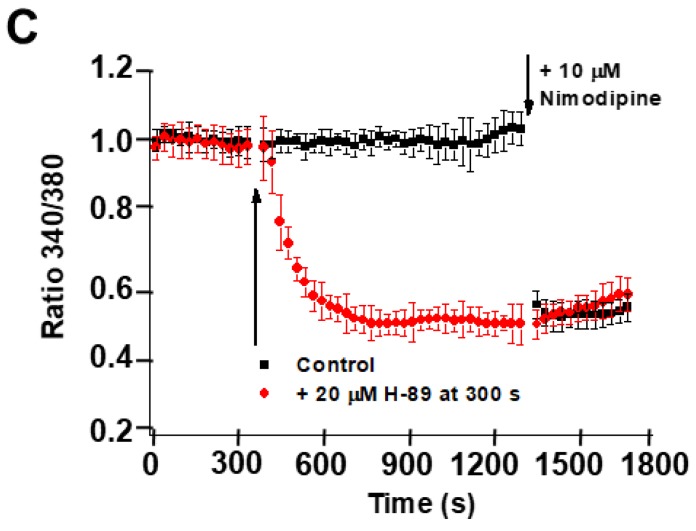
H-89 decreases CGN steady-state [Ca^2+^]_i_ to values obtained upon the blockade of LTCCs by nifedipine and nimodipine, while calphostin C has no effect. Mature CGNs in culture were loaded with Fura-2, as indicated in the Materials and Methods section, and then changed to MLocke’s K25 buffer (37 °C) and treated with the indicated protein kinase inhibitors. (**A**) Representative pseudocolor images of the same fields of selected CGN plates before (left-most column of images) and after 15 min incubation (middle column of images) with 20 µM of the PKA inhibitor (H-89) or in the absence of inhibitor (control) and 2 min after the addition of 10 µM nifedipine post-H-89 (images in the right-most column). Pseudocolor scale for 340/380 ratio values: light blue, 0.5–0.6; green, 1.0–1.1. Scale bar = 10 µm. The white-filled arrows pointing toward Panel B indicate the approximate times at which these images were captured. (**B**,**C**) Time dependence of population averages of the 340/380 ratio in neuronal somas before and after the addition of the indicated protein kinase inhibitors after 350 s (at the point indicated by the first arrow). The kinetic analysis of the 340/380 ratio of the somas of CGNs was done for controls (vehicle DMSO addition, solid black squares) and for the addition of 20 µM H-89 (red solid circles) and 2 µM calphostin C (blue down-triangles), inhibitors of PKA and PKC, respectively. Each inhibitor was added to a different culture plate. At approximately 1350 s (at the point indicated by the second arrow), 10 µM nifedipine (**B**) or 10 µM nimodipine (**C**) was added to the culture plates in all conditions. Data acquisition and analysis were done after the selection of neuronal somas using the region of interest (ROI) tool of the HCImage software, as indicated in the Materials and Methods section, with exposure times lower than 0.4 s at time intervals of 30 s. The 340/380 ratio values shown are the average ± S.E. of experiments done with three different preparations of CGNs (n > 300 neuronal somas of fields taken from six plates for each experimental condition).

Control experiments show that the 340/380 ratio of Fura-2-loaded CGNs in MLocke’s K25 is steady for at least 30 min, yielding an average 340/380 ratio of 1.0 ± 0.1 (Figure 5B), as also shown in previous works [9,34,47]. To evaluate the contribution of calcium entry through LTCCs to steady-state [Ca^2+^]_i_, we used the specific blockers nifedipine and nimodipine. The addition of either of these dihydropyridines produces a drop in the 340/380 ratio to a value of 0.55 ± 0.05 in less than 1 min after adding the LTCC blockers nifedipine or nimodipine (Figure 5). This is close to the steady-state 340/380 ratio measured after changing CGNs to the proapoptotic K5 medium, 0.50 ± 0.05 (see below), and this is consistent with the inactivation of LTCCs upon CGN plasma membrane polarization noticed in earlier works. PKA and PKC have been reported as possible modulators of LTCC activity, as they can affect Ca^2+^ influx through LTCCs [24,27]. As also shown in Figure 5, the addition of the PKA inhibitor H-89 decreases the 340/380 ratio to 0.55 ± 0.05. Notably, after 15 min of the addition of H-89, the 340/380 ratio reaches the steady-state value attained after the addition of the LTCC blockers nifedipine and nimodipine (Figure 5). These results show that the inhibition of PKA produces a potent inhibition of calcium entry through LTCCs in mature CGNs in MLocke’s K25 medium. In contrast, the addition of 2 μM of the PKC inhibitor calphostin C does not have a statistically significant effect on the steady 340/380 ratio, pointing out that in our experimental conditions, the PKC activity does not seem to be relevant to the control of the steady-state [Ca^2+^]_i_ in CGN soma.

These results lead to the conclusion that the effects of H-89, KN-93, and KN-92 on the steady-state [Ca^2+^]_i_ in the somas of CGN in MLocke’s K25 are largely dominated by the inhibition of calcium entry through LTCC.

### 2.5. Inhibition of PKA Alters the LTCC Response to Partial Depolarization of the Plasma Membrane in CGNs 

**Figure 6:**
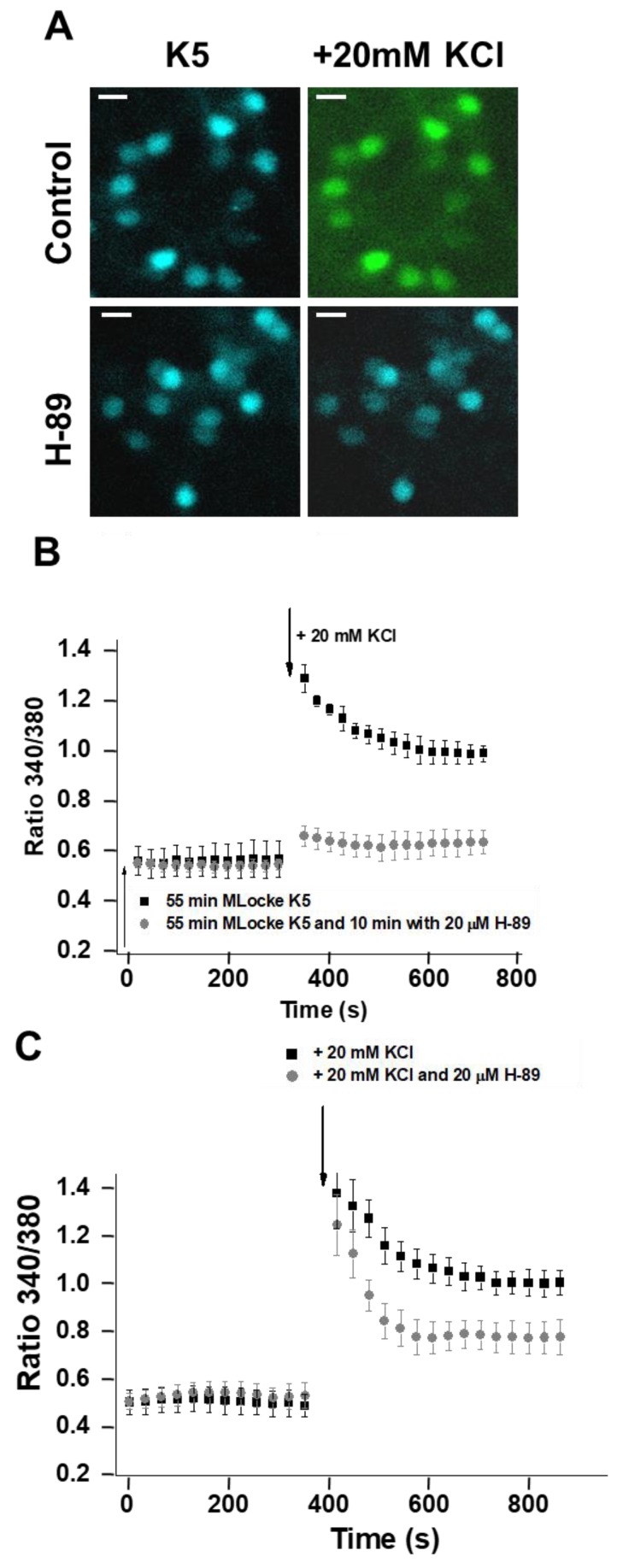
H-89 blocks the rise in [Ca^2+^]_i_ of CGNs in response to the increase in extracellular potassium. Mature CGNs in culture were loaded with Fura-2, as indicated in the Materials and Methods section, and then changed to MLocke’s K5 buffer (37 °C) and incubated for 1 h in a 5% CO_2_ culture chamber and thereafter treated as indicated below. (**A**) Representative pseudocolor images of the same fields of selected CGN plates after 1 h in low-potassium MLocke’s K5 before (left) and 10 min after the addition of a pulse of 20 mM KCl to the extracellular medium (right) for a control and after a 15 min incubation with 20 µM of the PKA inhibitor H-89. Pseudocolor scale for 340/380 ratio values: light blue, 0.5–0.6; green, 1.0–1.1. Scale bar = 10 µm. (**B**) Population averages of 340/380 ratio measurements in neuronal somas before and after the addition of 20 mM KCl at the time indicated with a black arrow. Before starting the measurements of 340/380 ratios, cell plates were incubated for 10 min with 20 µM of the PKA inhibitor (H-89, gray-filled circles). The results obtained for controls in the absence of these inhibitors (addition of the solvent DMSO used to solubilize H-89) are shown with solid black squares. (**C**) Population averages of 340/380 ratio measurements in neuronal somas before and after the addition of 20 mM KCl (control, solid black squares) or 20 mM KCl plus 20 µM H-89 (gray-filled circles) at the time indicated with a black arrow. For all the panels, each inhibitor was added to a different culture plate. Data acquisition and analysis were done after the selection of neuronal somas using the region of interest (ROI) tool of the HCImage software as indicated in the Materials and Methods section, with exposure times lower than 0.4 s at time intervals of 30 s. The 340/380 ratio values shown are the average ± S.E. of experiments done with three different preparations of CGNs (n > 300 neuronal somas of fields taken from six cell plates for each experimental condition).

In MLocke’s with 5 mM KCl, fluorescence imaging of CGNs loaded with Fura-2 yields a low steady 340/380 ratio in the neuronal somas of 0.50 ± 0.05, i.e., [Ca^2+^]_i_ ~40–50 nM (Figure 6A,B). The same 340/380 ratio readings were obtained in this medium in the presence or absence of H-89 (PKA inhibitor) added to the plate 10 min before starting the measurements of cytosolic calcium concentration (Figure 6A,B). However, H-89 largely impairs the kinetics of the [Ca^2+^]_i_ response to the addition of 20 mM KCl to the extracellular medium, which increases the extracellular KCl up to 25 mM. In the controls for CGNs loaded with Fura-2, the addition of 20 mM KCl elicits biphasic kinetics with a strong and fast rise in the 340/380 ratio until reaching values higher than 1.0—between 1.2 and 1.5 for most neuronal somas in the plates—followed by slower kinetics for the decay in the 340/380 ratio (t½ ≤ 2 min) until reaching values of approximately 0.95 ± 0.05 (Figure 6A,B), i.e., [Ca^2+^]_i_~150 ± 30 nM, which is within the range obtained for the somas of CGNs loaded with Fura-2 several minutes after changing the cells to MLocke’s K25 [34]. The pre-incubation of CGNs with H-89 for only 15 min largely attenuates the rise in the 340/380 ratio after the addition of 20 mM KCl to the extracellular medium (Figure 6A,B). Noteworthy is that the steady-state 340/380 ratio attained after several minutes reaches a value of 0.60 ± 0.05, which is very close to the (340/380) ratio measured for the somas of control CGN plates before the addition of 20 mM KCl to the extracellular MLocke’s medium. The blockade of the [Ca^2+^]_i_ response by the inhibitor of PKA requires several minutes of CGN pre-incubation, since only a slight attenuation of the strong and fast rise in the 340/380 ratio is observed when the PKA inhibitor is added simultaneously with 20 mM KCl to the extracellular MLocke’s medium (Figure 6C). This correlates with the change in the phosphorylation level of LTCCs elicited by the PKA inhibitor H89 after only a 15 min pre-incubation (Figure 3). It is to be noted that a 15 min pre-incubation of Fura-2-loaded CGNs with 2 µM of the PKC inhibitor calphostin C before the addition of 20 mM KCl has, at most, a weak effect that is statistically nonsignificant on the kinetics of the [Ca^2+^]_i_ response to the addition of 20 mM KCl to the extracellular medium.

## 3. Discussion

The results of this work show that lipid rafts allow for more efficient control of the LTCC phosphorylation level in mature CGNs in vitro. Here, it is shown that PKA and CaMK-II also associate with caveolin-1-rich lipid rafts in the plasma membrane of CGN and that treatment with the lipid raft-disrupting agent MβCD largely decreases the phosphorylation level of the β_2_ subunit of LTCCs to levels observed in the presence of inhibitors of PKA and CaMK-II, and also to those observed in the proapoptotic conditions attained by low potassium (5 mM) in the extracellular medium, an experimental condition that inactivates LTCCs [33]. These effects of MβCD are observed at concentrations that induce a large depletion of cholesterol in CGNs and before a significant loss of cell viability. Consistently, the pretreatment of CGNs with MβCD and the removal of MβCD–cholesterol complexes formed results in a decrease in the steady-state cytosolic [Ca^2+^]_i_ in the neuronal somas; the decrease is close to that induced by the LTCC blocker nifedipine, highlighting that MβCD treatment leads to a large functional inactivation of LTCCs, even in the partial depolarizing conditions elicited by 25 mM KCl in the extracellular medium. Taken together, our results strongly support the notion that the disruption of lipid rafts impairs the activation of LTCCs by partial depolarizing conditions by decreasing the phosphorylation of their β_2_ subunit by PKA and CaMK-II. Indeed, it is to be noted that the initial rate and kinetic half-time of the decrease in [Ca^2+^]_i_ produced by the CaMK-II inhibitor KN-93 are about 3-fold higher than those obtained for the noninhibitor analogue KN-92 (Figure 3C), despite both displaying a potency similar as inhibitors of LTCC [46]. Moreover, a difference of ~2 min between the half-time values of the kinetics of the decrease in [Ca^2+^]_i_ by KN-92 and KN-93 can be accounted for by the well-known slow kinetics of the loss of calcium-independent CaMK-II activity, which has been reported to take place at a timescale of minutes in neurons [50]. As we showed in a previous work that functional lipid rafts define the high-calcium sub-microcompartments near the plasma membrane of mature CGNs [9], these results are also consistent with a stringent requirement for local Ca^2+^-dependent activation of CaMK-II in the channel vicinity, as suggested by the blockade of CaMK-II-dependent LTCC facilitation by the “fast” Ca^2+^ chelator 1,2-*bis*(2-aminophenoxy)ethane-*N*,*N*,*N*′,*N*′-tetraacetic acid (BAPTA) but not by the slow chelator ethylene glycol-*bis*(β-aminoethyl ether) *N*,*N*,*N*′,*N*′-tetraacetic acid (EGTA) [51]. In addition, our results are in accordance with a previous work reporting the modulation of the structural coupling between LTCCs and regulatory proteins by cholesterol in cardiomyocytes [52], where the authors concluded that alterations in the current of LTCCs are mediated by MβCD.

The relevance of the sustained increase in [Ca^2+^]_i_ produced by 25 mM KCl plasma membrane depolarization to CGN survival is highlighted by the fact that, within the first 3 h after inducing apoptosis in 5 mM KCl medium, cell death can be largely blocked by simply increasing the KCl concentration of the extracellular medium to 25 mM [18,48], which promotes neuronal survival. The results of this work show that in mature CGN cultures, the level of phosphorylation of the β_2_ subunit of LTCCs in a survival medium containing 25 mM KCl is significantly higher than that in proapoptotic 5 mM KCl medium, thus uncovering a major role for the phosphorylation of the LTCC β_2_ subunit in the activity of these calcium channels. Indeed, our results show that LTCC activity accounts for nearly 85% of the steady-state increase in cytosolic calcium concentration in the CGN somas that is observed with the partial depolarization of the plasma membrane when extracellular potassium rises from 5 mM (proapoptotic condition) to 25 mM (neuron survival condition) (see the Supplementary Figure). This is in agreement with the major relevance of LTCC activity to the survival of these neurons in culture which was noticed earlier [33,34,38], and it is also consistent with the low level of l-glutamate detected in the extracellular medium when CGNs are in 25 mM KCl MLocke’s medium [35]. Treatment of CGNs in 25 mM KCl MLocke’s medium with the PKA inhibitor H-89 decreases the steady-state cytosolic [Ca^2+^]_i_ down to the values measured in the proapoptotic 5 mM KCl MLocke’s medium in less than 15 min. Moreover, the effects of H-89 on the steady-state [Ca^2+^]_i_ in the somas of CGNs in MLocke’s K25 are largely dominated by the inhibition of calcium entry through LTCCs. Noteworthy is that the sum of the decreases in the phosphorylation level of the LTCC β_2_ subunit elicited by CaMK-II and PKA inhibitors correlates with the large decrease in the phosphorylation level of the LTCC β_2_ subunit resulting from the proapoptotic conditioning of CGNs. Thus, the phosphorylation of the β_2_ subunit can largely account for the activation of LTCCs by partial plasma membrane depolarization induced by the presence of 25 mM KCl in the extracellular medium. In contrast, we found that the steady-state cytosolic [Ca^2+^]_i_ is not significantly altered by calphostin C, an inhibitor of PKC, excluding a major role for this protein kinase in the control of cytosolic calcium homeostasis in mature CGNs in culture. 

Our results also indicate that the activity of these calcium channels plays the leading role in modulating the excitability threshold of CGNs. Under our experimental conditions in culture and in the absence of neurotrophic factors, the contribution of other calcium channels and of NMDAr to maintain a sustained level of cytosolic [Ca^2+^]_i_ and thus promoting neuronal survival is only very minor for mature CGNs [34,35] (see also the Supplementary Figure). In the brain, nearly 80% of the α-subunits of LTCCs belong are the Ca_V_1.2 (α_1C_) subtype, and 10–25% are the subtype Ca_V_1.3 (α_1D_) [53], while in cerebellar granule neurons, Ca_V_1.2 accounts for 89% and Ca_V_1.3 for 11% of LTCC transcripts [54]. Interestingly, the hyperactivation of Ca_V_1.2 LTCCs by CaMK-II is implicated in Timothy Syndrome, a multiorgan human genetic disorder whose symptoms include mental retardation and cardiac disease [55,56], and the excessive activation of Ca_V_1.3 LTCCs is implicated in the loss of dendritic spines following dopamine depletion in animal models of parkinsonism [57]. Thus, the activity of LTCCs plays a major role in the fine-tuning of CGN excitability, as cytosolic [Ca^2+^]_i_ plays a major role in neuronal secretory activity—both the basal secretory activity and the minimum stimuli needed to elicit a neuronal response. Indeed, it has been shown that LTCCs play a relevant role in NMDAr-independent long-term potentiation [58]. Therefore, the impairment of LTCC activity by MβCD is likely to be a molecular mechanism underlying the neurological disorders induced by this compound.

The altered LTCC response to a partial depolarization of the plasma membrane in CGNs upon inhibition of PKA, as reported in this work, lends support to a major role for this protein kinase in the normal functioning of granule neurons in the cerebellar cortex. Several β subunits of LTCCs are expressed in rat brain cerebellum [59], namely, β_2_, β_3_, and β_4_, but not the isoform β_1_, with the β_4_ isoform of the β subunit showing higher levels of expression. However, it has been shown that activated/autophosphorylated CaMK-II binds to the β_2_ isoform, but it does not bind to β_3_ or to β_4_ [27]. Also, CaMK-II coimmunoprecipitates with forebrain LTCCs that contain Ca_V_1.2α_1_ and β_1_ or β_2_ subunits, but CAMK-II is not detected in LTCC complexes containing β_4_ subunits [60]. In addition, it has been demonstrated that LTCC modulation by PKA is due to β_2_ and α_1c_ subunit phosphorylation [23,24]. Mutations of serine 478 (Ser478) and serine 479 (Ser479) from the β_2_ subunit resulted in the complete inhibition of the PKA-induced increase in calcium currents through LTCCs [23]. On these grounds, we selected for this study an antibody produced by FabGennix Inc. (PCCb2-140AP), using as the immunogen a synthetic peptide with the amino acid sequence “ecs kqr s_(p)_rh kskdry c” which is located near the C-terminal end of the rat β_2_ subunit of LTCCs and which is absent in β_3_ and β_4_ isoforms. This amino acid sequence is also very close to other phosphorylation sites reported for PKA and CaMK-II in the β_2_ subunit of LTCCs, namely, Ser478, Ser479, and Thr498 [23,26,27]. Moreover, the two serines present in this sequence, which correspond to Ser566 and Ser570 in rat’s β_2_ subunit (Uniprot: Q8VGC3), are also potential sites for phosphorylation by both kinases, particularly Ser570, which fulfills the criteria for a consensus phosphorylation site of CaMK-II. Indeed, Grueter et al. [27] reported a stoichiometry of 6 phosphorylation sites targeted by CaMK-II per mol of β_2_ subunits. Thus, our results can be rationalized in terms of phosphorylation of the β_2_ subunit of LTCCs at the serines present in the amino acid sequence “ecs kqr s_(p)_rh kskdry c” or in terms of immunoreactivity of the PCCb2-140AP antibody with the vicinal phosphorylation sites, i.e., Ser478_(P)_ and/or Ser479_(P)_ and/or Thr498_(P)_. It is to be noted that both CaMK-II and PKA have been reported to act synergistically to increase the calcium current intensity through LTCCs in cardiac myocytes [61].

**Figure 7:**
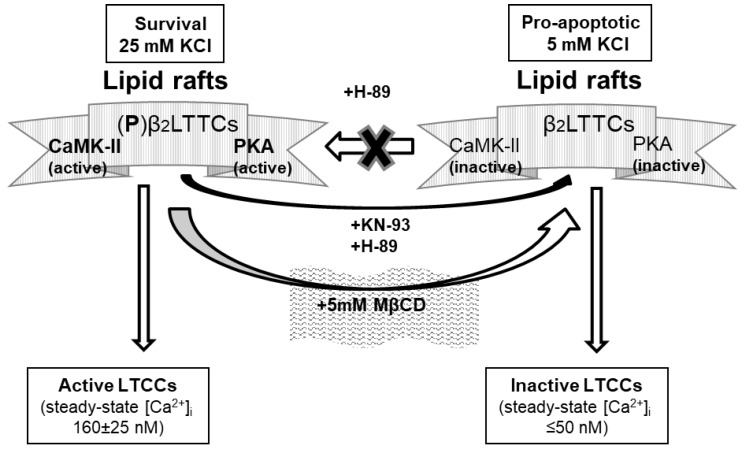
Schematic diagram summarizing the major results and conclusions of this work. The rise in the steady-state [Ca^2+^]_i_ in neuronal somas up to the values needed for CGN survival induced by the increase in extracellular K^+^ from 5 to 25 mM is mediated by CaMK-II and PKA phosphorylation of the β_2_ subunit of LTCCs clustered in caveolin-1-rich lipid rafts. The gray contour filled with thin vertical lines represents caveolin-1-rich lipid rafts containing LTCC, CaMK-II, and PKA. The inhibitors of CaMK-II (KN-93) and of PKA (H-89) and the lipid raft-disrupting compound MβCD decrease the levels of phosphorylated LTCC, (P)-β_2_LTCC, to the values measured in proapoptotic 5 mM KCl medium, resulting in the inactivation of LTCCs and a sustained proapoptotic low steady-state [Ca^2+^]_i_ in neuronal somas. For the sake of simplicity, other proteins present in caveolin-1-rich lipid rafts of mature CGNs (see, e.g., [8,9,10,49,62]) are not included in this schematic diagram.

In conclusion, the results of this work reveal a critical role for the phosphorylation of the β_2_ subunit of LTCCs by the CaMK-II and PKA associated with caveolin-1-rich lipid rafts to maintain LTCC activity in CGNs grown in culture. In addition, our results show that cholesterol depletion by MβCD decreases the steady-state [Ca^2+^]_i_ down to the sustained proapoptotic low [Ca^2+^]_i_ range. This is summarized in the scheme drawn in Figure 7. Whether pharmacological overstimulation of PKA or CaMK-II can counteract the decrease in the phosphorylation of the LTCC β_2_ subunit and the decrease in steady-state [Ca^2+^]_i_ upon partial lipid raft disruption remain to be settled. 

## 4. Materials and Methods

### 4.1. Preparation of Rat Cerebellar Granule Neurons (CGNs)

CGNs were obtained from dissociated cerebella of 7-days-old Wistar rats as described previously [8,9,34,47,48,49,62,63]. All animal handling was performed in accordance with Spanish regulations and approved by the Ethical Committee of the University of Extremadura. Briefly, cells were plated in Dulbecco’s Modified Eagle medium (DMEM) supplemented with 10% heat-inactivated fetal bovine serum, 5 mM glucose, 19.63 mM KCl, 3.7 ng/mL insulin, 7 μM 4-aminobenzoic acid, 50 U/mL penicillin, 25 U/mL streptomycin, 0.91 mM pyruvate, and 2 mM glutamine on 35 mm diameter dishes (Corning, NY, USA) coated with poly-d-lysine at a density of 2.5 × 10^6^ cells/dish. Cultures were maintained at 37 °C in a humidified atmosphere of 95% air/5% CO_2_. Cytosine arabinofuranoside (10 μM) was added to fresh culture medium 48 h after plating to prevent the replication of non-neuronal cells. Seven days after plating, the culture medium was replaced with the serum-free DMEM:F12 medium (1:1) supplemented with 12.5 mM glucose, 20.82 mM KCl, 5 μg/mL insulin, 0.1 mg/mL apo-transferrin, 20 nM progesterone, 50 U/mL penicillin, 25 U/mL streptomycin, 0.1 mg/mL pyruvate, and 2 mM l-glutamine. Mature CGNs at 9–10 days in vitro were used in all the experiments.

Buffer composition used in this work is as follows: (a) MLocke’s K25 buffer (pH 7.4 at 37 °C): 4 mM NaHCO_3_, 10 mM Tricine, 5 mM glucose, 2.3 mM CaCl_2_, 1 mM MgCl_2_, and 134 mM NaCl/25 mM KCl; (b) MLocke’s K5 buffer (pH 7.4 at 37 °C): 4 mM NaHCO_3_, 10 mM Tricine, 5 mM glucose, 2.3 mM CaCl_2_, 1 mM MgCl_2_, and 154 mM NaCl/5 mM KCl.

Cell viability was experimentally assessed by measuring the amount of colored formazan by the reduction of 3-(4,5-dimethylthiazol-2-yl)-2,5-diphenyltetrazolium bromide (MTT) as in previous works [9,34,47,48,63].

### 4.2. Measurement of the Intracellular Free Ca^2+^ Concentration ([Ca^2+^]_i_)

[Ca^2+^]_i_ was measured as in previous works [9,15,34,35,47,63]. Briefly, CGNs were loaded with Fura-2 by incubation for 60 min in DMEM-F12 containing, unless stated otherwise, 5 µM Fura-2-acetoxymethyl ester (Fura-2 AM) and 0.025% Pluronic-F127 at 37 °C. Afterward, CGNs were washed and the culture dish placed in a thermostatically controlled plate (Warner Instrument Co., Hamden, CT, USA) of a Nikon Diaphot 300 (Tokyo, Japan) inverted microscope, equipped with an epifluorescence attachment and excitation filter wheel. Digital images with 340 and 380 nm excitation filters were taken with a Hamamatsu Orca-R2 CCD camera (binning mode 2 × 2) and Lamdba 10–2 filter wheel controller, and images were subsequently analyzed with HCImage software. Data acquisition and analysis were done after the selection of the neuronal somas using the region of interest (ROI) tool of this software. The data of 340/380 ratios are presented as population averages, and the total number of neuronal somas averaged in each experimental condition are indicated in the legends for the figures. A value of 224 nM was used for the dissociation constant of the Fura-2/Ca^2+^ complex to obtain the [Ca^2+^]_i_ values reported in this work, as in previous works [34,35].

### 4.3. CGN Cell Lysates and Western Blotting

Cell lysates used for controls and specific treatments were obtained from CGN cultures after a 2000× *g* centrifugation for 2 min at 4 °C in a refrigerated Eppendorf microcentrifuge followed by pellet resuspension in lysis buffer (25 mM Tris–HCl, pH 7.4, 150 mM NaCl, 5 mM EDTA, 50 mM NaF, 5 mM NaVO_3_, and 0.25% Triton X-100, supplemented with the Roche Biochemicals protease inhibitor cocktail COMPLETE). Using Bradford’s method, we determined the protein concentration of cell lysates and samples for running on an SDS-PAGE gel for Western blotting analysis.

In an SDS-PAGE gel at a concentration of 7.5, 10.4, or 12% acrylamide, for the same protein of interest, a certain amount of CGN lysate or lipid raft fraction was loaded per lane, ranging between 5 and 20 μg of protein in different gels; then, we transferred the gel to PVDF or nitrocellulose membranes with a 0.2 and 0.45 μm average pore size, respectively (Trans-BloT TransferMedium, BioRad, Hercules, CA, USA). Depending on the protein of interest, membrane blocking was carried out with 5% (*w*/*v*) non-fat dry milk or 5% bovine serum albumin, both in phosphate-buffered saline (PBS) supplemented with 0.05% polyoxyethylenesorbitan monolaurate (PBST). Before incubation with the primary antibody, membranes were washed three times with PBST. The immunodetection of proteins was performed with their specific primary antibody at a dilution of 1:100 in PBST. After incubation with the first antibody overnight, membranes were washed six times with PBST and incubated for 1 h at room temperature with the secondary IgG antibody conjugated with horseradish peroxidase. Secondary anti-rabbit IgG–horseradish peroxidase (Sigma A0545, Sigma-Aldrich, St. Louis, MI, USA) or anti-mouse IgG–horseradish peroxidase (Pierce-1858413) was used at a dilution of 1:25,000 and 1:5000 in PBST, respectively. Again, we washed the membrane six times with PBST followed by incubation for 3 min with Super-Signal West Dura Substrate (Pierce). Western blots were revealed by exposure to an Amersham Hyperfilm MP autoradiography film (GE Healthcare, UK) or with Bio-Rad ChemiDoc^TM^ XRS+. Then, membranes were treated under continuous stirring at room temperature with the following stripping buffers: (1) 10 min with 0.2 M glycine/0.5 M NaCl brought to pH 2.8 with acetic acid and (2) 10 min with 0.5 M acetic acid/0.5 M NaCl at pH 2.5. After washing with distilled water for 10 min, membranes were blocked with 3% bovine serum albumin in PBST and treated as indicated above to quantify β-actin to monitor the protein load, using mouse anti-β-actin (Sigma-Aldrich-A1978, 1:100 dilution) as the primary antibody and anti-mouse IgG–horseradish peroxidase (Pierce-1858413, 1:5000 dilution) as the secondary antibody.

### 4.4. Lipid Raft Preparation

CGN lipid rafts were isolated as indicated in previous works [8,9,10], except that all the buffers were supplemented with the phosphatase inhibitors NaF and NaVO_3_. Briefly, sucrose was added to cell lysates to obtain a 41% concentration and then loaded over two layers of sucrose: 125 μL of 35% sucrose and 500 μL of 16% sucrose, prepared in 25 mM Tris–HCl, pH 7.4, 150 mM NaCl, 5 mM EDTA, 50 mM NaF, and 5 mM NaVO_3_. For lipid raft isolation, samples were centrifuged for 5 h at 4 °C in an SW60 rotor at 256,000× g for the average radius, and 10 fractions were collected (from the top to the bottom, fractions 1–10). Protein concentration was determined using the Bradford protein assay. Samples were analyzed by Western blotting as described above.

### 4.5. Immunoprecipitation

Immunoprecipitation was carried out using the ImmunoCruz^TM^ IP/WB Optima system of Santa Cruz Biotechnology Inc. (Santa Cruz, CA, USA) following the instructions given in their technical data sheets. Briefly, complexes between the matrix and antibodies were prepared as follows. In an Eppendorf tube, 5 µg of anti-caveolin-1 (sc-894) was mixed with 50 µL of the appropriate matrix (IgG rabbit) and 500 µL of Tris-buffered saline. After 1 h incubation in a tube-rotor with continuous shaking, the matrix was precipitated by 30 s centrifugation at 4 °C at 16,100× *g* in a refrigerated Eppendorf microcentrifuge 5415R. The supernatant was removed, and the precipitated matrix was subjected to three washes with 500 µL of Tris-buffered saline by 30 s centrifugation at 4 °C at 16,100× *g* in a refrigerated Eppendorf microcentrifuge in each washing step. Before immunoprecipitation, the cell lysates of CGNs were centrifuged at 500× *g* for 10 min to remove nuclei and cell debris. Then, cell lysates were treated with 1% Triton-X100 for 30 min with mild stirring, and 400 µg of protein lysate was added to the tube containing the matrix/antibody complex, prepared as indicated above, and incubated overnight at 4 °C with continuous shaking in a tube-rotor. The matrix was precipitated by 30 s centrifugation at 16,100× *g* and 4 °C, and the supernatant was carefully removed. The precipitated matrix was resuspended in Tris-buffered saline and centrifuged again at 16,100× *g* and 4 °C for a more complete removal of the remaining supernatant. This washing step was repeated three times. Supernatants and washed matrix precipitate were mixed with standard sample buffer for SDS-electrophoresis, boiled for 5 min, and loaded onto SDS-PAGE gels. Samples were analyzed by SDS-PAGE followed by Western blotting as indicated above.

### 4.6. Measurement of Cholesterol Content in Cell Lysates

To quantify the extent of cholesterol removal by treatment with MβCD, we treated CGNs with 0, 5, 10, and 20 mM MβCD for 15 min in MLocke’s K25 buffer. After the treatment, the supernatant was removed, and CGNs were carefully lysed, as indicated above. Cell lysates were homogenized by 50–60 passages through hypodermic needles, stainless steel gauge 26 of ½ inch length. The protein content of these cell lysates was measured using the Bradford assay, and their cholesterol content was measured using a standard assay with Amplex Red^TM^ (Invitrogen Life Sciences Technologies –Thermo Fisher Scientific). The fluorescent product of oxidation of Amplex Red^TM^, resorufin, was measured with a Perkin-Elmer 650–40 fluorimeter operated in ratio mode, with a fixed excitation wavelength at 530 nm and emission wavelength at 590 nm, with 10/10 excitation/emission slits. Amplex Red 50 μM was added to the assay buffer (pH 7.0): 2-{[2-hydroxy-1,1-*bis*(hydroxymethyl) ethyl]amino} ethanesulfonic acid (TES) 50 mM, NaCl 100 mM, EDTA 0.1 mM in the presence of horseradish peroxidase 0.2 U/mL. Cholesterol oxidase 1 U/mL was added, and fluorescence was recorded for 20 min at 25 °C until the intensity reached a plateau.

### 4.7. CGN Treatments

In all experiments with proapoptotic low-potassium conditions, CGN plates were treated with MLocke’s K5 buffer for 1 h at 37 °C, 5% CO_2_. In experiments performed in low potassium, CGN plates loaded with Fura-2 AM were incubated for 15 min with H-89 (PKA inhibitor) at 37 °C, 5% CO_2_, before image acquisition for [Ca^2+^]_i_ measurements.

To study the phosphorylation levels of the β_2_ subunits of LTCC after protein kinase inhibition, we treated CGN plates for 15 min at 37 °C and 5% CO_2_ with 20 µM H-89 to inhibit PKA and 30 µM KN-93 to inhibit CaMK-II. These plates were lysed as previously described.

To study the effect of cholesterol extraction on the phosphorylation levels of LTCCs, CGNs were treated for 15 min with 1 mM and 5 mM methyl-β-cyclodextrin (MβCD), a well-known cell-cholesterol sequestering compound, in MLocke’s K25 buffer. MβCD concentrations were selected taking into account our previous studies of cell viability [11]. Finally, to study the effect of cholesterol extraction on [Ca^2+^]_I_, CGNs were treated with 5 mM MβCD in serum-free DMEM:F12 medium (1:1), supplemented as indicated before, and, after 1 h treatment, CGNs were washed and loaded with Fura-2 AM for measurements of [Ca^2+^]_i_. In this latter case, the incubation of CGNs with MβCD could not be carried out simultaneously with Fura-2 AM nor with preloaded cells, since the treatment of CGNs for 30–45 min with MβCD produces a large release of Fura-2 from CGNs to the extracellular medium.

### 4.8. Chemicals and Reagents

Primary antibodies: rabbit anti-LTCC α_1C_ subunit (sc-25686), rabbit anti-rabbit PKA (sc-28892), rabbit anti-CaMK-II (sc-9035), and rabbit anti-caveolin-1 (sc-894) were supplied by Santa Cruz Biotechnology (Santa Cruz, CA, USA);. rabbit anti-p-β_2_ subunit of LTCCs (PCCb2-140AP) was supplied by FabGennix Inc. (Frisco, TX, USA); and mouse anti-β-actin (A1978) was supplied by Sigma-Aldrich (Spain office). All these antibodies were used in the dilution range recommended in their technical sheets and tested for the detection of molecular weight bands expected for their corresponding proteins with whole CGN lysates before use in the experiments of this work. Western blots reagents and anti-rabbit IgG–horseradish peroxidase was supplied by Sigma-Aldrich, and anti-mouse IgG–horseradish peroxidase and SuperSignal West Dura Extended Duration Substrate were supplied by Pierce (Rockford, IL, USA). KB-7943 and ω-conotoxin MVIIC were purchased from Tocris Bioscience (Bristol, UK). MβCD, MK-801, nifedipine, nimodipine, and the specific protein kinase inhibitors H-89 (B1427), KN-93 (K1385), and calphostin C (C6303) were supplied by Sigma-Aldrich (Spain office). The ImmunoCruz^TM^ IP/WB Optima B system (sc-45039) and KN-92 (sc-311369) were purchased from Santa Cruz Biotechnology (Santa Cruz, CA, USA). Fura-2 AM and Amplex Red^TM^ from Invitrogen Life Sciences Technologies were supplied by Thermo Fisher Scientific (Spain office). All other reagents and chemicals were of analytical grade from Sigma-Aldrich (Spain office) or Roche-Merck (Darmstadt, Germany).

### 4.9. Statistical Analysis

Results are expressed as the mean ± standard error (S.E.). Statistical analysis was carried out by the Mann–Whitney non-parametric test. A significant difference was accepted at the *p* < 0.05 level. All the results were confirmed with duplicate measurements of at least three different CGN preparations.

## Figures and Tables

**Table 1:** A short-term incubation with methyl-β-cyclodextrin (MβCD) efficiently extracts cholesterol from cerebellar granule neurons (CGNs) while preserving cell viability.

[MβCD] (mM)	Cholesterol Content (nmol/mg of CGN Protein)	Cell Viability
0	22.8 ± 2.1	100 ± 5%
5	8.4 ± 0.9	100 ± 5%
10	4.6 ± 0.4	100 ± 6%
20	4.7 ± 0.4	95 ± 5%

## Supplementary Materials

Supplementary Materials can be found at http://www.mdpi.com/1422-0067/19/11/3667/s1.

## Abbreviations

[Ca^2+^]_i_	intracellular free calcium concentration
CaMK-II	calcium/calmodulin-dependent protein kinase II
CGN	cerebellar granule neurons
DMEM	Dulbecco’s modified Eagle medium
EDTA	ethylenediamine tetraacetic acid
Fura-2 AM	Fura-2-acetoxymethyl ester
LTCC	L-type calcium channels
MβCD	methyl-β-cyclodextrin
NMDAr,	*N*-methyl d-aspartate receptor
PBS	phosphate-buffered saline
PBST	PBS supplemented with 0.05% polyoxyethylenesorbitan monolaurate (Tween 20)
PKA	protein kinase A
PKC	protein kinase C
VGCCs	voltage-gated calcium channels.

## References

[1] Tiwari G., Tiwari R., Rai A.K. (2010). Cyclodextrins in delivery systems: Applications. J. Pharm. Bioallied Sci..

[2] Shelley R.H., Babu J. (2018). Role of Cyclodextrins in Nanoparticle-Based Drug Delivery Systems. J. Pharm. Sci..

[3] López-Nicolás J.M., Rodríguez-Bonilla P., García-Carmona F. (2014). Cyclodextrins and Antioxidants. Crit. Rev. Food Sci. Nutr..

[4] Crumling M.A., King K.A., Duncan R.K. (2017). Cyclodextrins and Iatrogenic Hearing Loss: New Drugs with Significant Risk. Front. Cell. Neurosci..

[5] Barman S., Nayak D.P. (2007). Lipid Raft Disruption by Cholesterol Depletion Enhances Influenza A Virus Budding from MDCK Cells. J. Virol..

[6] Lu J.-C., Chiang Y.-T., Lin Y.-C., Chang Y.-T., Lu C.-Y., Chen T.-Y., Yeh C.-S. (2016). Disruption of Lipid Raft Function Increases Expression and Secretion of Monocyte Chemoattractant Protein-1 in 3T3-L1 Adipocytes. PLoS ONE.

[7] Ulloth J.E., Almaguel F.G., Padilla A., Bu L., Liu J.-L., De Leon M. (2007). Characterization of methyl-β-cyclodextrin toxicity in NGF-differentiated PC12 cell death. NeuroToxicology.

[8] Marques-da-Silva D., Gutierrez-Merino C. (2012). L-type voltage-operated calcium channels, N-methyl-d-aspartate receptors and neuronal nitric-oxide synthase form a calcium/redox nano-transducer within lipid rafts. Biochem. Biophys. Res. Commun..

[9] Marques-da-Silva D., Gutierrez-Merino C. (2014). Caveolin-rich lipid rafts of the plasma membrane of mature cerebellar granule neurons are microcompartments for calcium/reactive oxygen and nitrogen species cross-talk signalling. Cell Calcium.

[10] Marques-da-Silva D., Samhan-Arias A.K., Tiago T., Gutierrez-Merino C. (2010). L-type calcium channels and cytochrome *b*_5_ reductase are components of protein complexes tightly associated with lipid rafts microdomains of the neuronal plasma membrane. J. Proteomics.

[11] Catterall W.A. (2000). Structure and regulation of voltage-gated Ca^2+^ channels. Annu. Rev. Cell Dev. Biol..

[12] Dolphin A.C. (2003). Beta subunits of voltage-gated calcium channels. J. Bioenerg. Biomembr..

[13] Buraei Z., Yang J. (2013). Structure and function of the β subunit of voltage-gated Ca²⁺ channels. Biochim. Biophys. Acta.

[14] Hohaus A., Person V., Behlke J., Schaper J., Morano I., Haase H. (2002). The carboxyl-terminal region of AHNAK provides a link between cardiac L-type Ca^2+^ channels and the actin-based cytoskeleton. FASEB J..

[15] Tiago T., Marques-da-Silva D., Samhan-Arias A.K., Aureliano M., Gutierrez-Merino C. (2011). Early disruption of the actin cytoskeleton in cultured cerebellar granule neurons exposed to 3-morpholinosydnonimine-oxidative stress is linked to alterations of the cytosolic calcium concentration. Cell Calcium.

[16] Razani B., Rubin C.S., Lisanti M.P. (1999). Regulation of cAMP-mediated Signal Transduction via Interaction of Caveolins with the Catalytic Subunit of Protein Kinase A. J. Biol. Chem..

[17] Davare M.A., Dong F., Rubin C.S., Hell J.W. (1999). The A-kinase anchor protein MAP2B and cAMP-dependent protein kinase are associated with class C L-type calcium channels in neurons. J. Biol. Chem..

[18] Gallo V., Kingsbury A., Balázs R., Jørgensen O.S. (1987). The role of depolarization in the survival and differentiation of cerebellar granule cells in culture. J. Neurosci..

[19] Gardoni F., Bellone C., Cattabeni F., Di Luca M. (2001). Protein Kinase C Activation Modulates α-Calmodulin Kinase II Binding to NR2A Subunit of N-Methyl-D-Aspartate Receptor Complex. J. Biol. Chem..

[20] Li Y., Zhang X., Liu H., Cao Z., Chen S., Cao B., Liu J. (2012). Phosphorylated CaMKII post-synaptic binding to NR2B subunits in the anterior cingulate cortex mediates visceral pain in visceral hypersensitive rats. J. Neurochem..

[21] Suzuki T., Du F., Tian Q.-B., Zhang J., Endo S. (2008). Ca^2+^/calmodulin-dependent protein kinase IIα clusters are associated with stable lipid rafts and their formation traps PSD-95. J. Neurochem..

[22] Pinard C.R., Mascagni F., McDonald A.J. (2005). Neuronal localization of Cav1.2 L-type calcium channels in the rat basolateral amygdale. Brain Res..

[23] Bünemann M., Gerhardstein B.L., Gao T., Hosey M.M. (1999). Functional Regulation of L-type Calcium Channels via Protein Kinase A-mediated Phosphorylation of the β_2_ Subunit. J. Biol. Chem..

[24] Kamp T.J., Hell J.W. (2000). Regulation of Cardiac L-Type Calcium Channels by Protein Kinase A and Protein Kinase C. Circ. Res..

[25] Hudmon A., Schulman H., Kim J., Maltez J.M., Tsien R.W., Pitt G.S. (2005). CaMKII tethers to L-type Ca^2+^ channels, establishing a local and dedicated integrator of Ca^2+^ signals for facilitation. J. Cell Biol..

[26] Lee T.S., Karl R., Moosmang S., Lenhardt P., Klugbauer N., Hofmann F., Kleppisch T., Welling A. (2006). Calmodulin kinase II is involved in voltage dependent facilitation of the L-type Cav1.2 calcium channel: Identification of the phosphorylation sites. J. Biol. Chem..

[27] Grueter C.E., Abiria S.A., Wu Y., Anderson M.E., Colbran R.J. (2008). Differential regulated interactions of calcium/calmodulin-dependent protein kinase II with isoforms of voltage-gated calcium channel beta subunits. Biochemistry.

[28] Schengrund C.-L. (2010). Lipid rafts: Keys to neurodegeneration. Brain Res. Bull..

[29] Sebastião A.M., Colino-Oliveira M., Assaife-Lopes N., Dias R.B., Ribeiro J.A. (2013). Lipid rafts, synaptic transmission and plasticity: Impact in age-related neurodegenerative diseases. Neuropharmacology.

[30] Sonnino S., Aureli M., Grassi S., Mauri L., Prioni S., Prinetti A. (2014). Lipid rafts in neurodegeneration and neuroprotection. Mol. Neurobiol..

[31] Di Paolo G., Kim T.-W. (2011). Linking Lipids to Alzheimer’s Disease: Cholesterol and Beyond. Nat. Rev. Neurosci..

[32] Berridge M.J., Lipp P., Bootman M.D. (2000). The versatility and universality of calcium signalling. Nat. Rev. Mol. Cell Biol..

[33] Franklin J.L., Johnson E.M. (1992). Suppression of programmed neuronal death by sustained elevation of cytoplasmic calcium. Trends Neurosci..

[34] Gutierrez-Martin Y., Martin-Romero F.J., Henao F., Gutierrez-Merino C. (2005). Alteration of cytosolic free calcium homeostasis by SIN-1: High sensitivity of L-type Ca^2+^ channels to extracellular oxidative/nitrosative stress in cerebellar granule cells. J. Neurochem..

[35] Garcia-Bereguiain M.A., Samhan-Arias A.K., Martin-Romero F.J., Gutierrez-Merino C. (2008). Hydrogen sulfide raises cytosolic calcium in neurons through activation of L-type Ca^2+^ channels. Antioxid. Redox Signal..

[36] Marchetti C., Usai C. (1996). High affinity block by nimodipine of the internal calcium elevation in chronically depolarized rat cerebellar granule neurons. Neurosci. Lett..

[37] Evans G.J., Pocock J.M. (1999). Modulation of neurotransmitter release by dihydropyridine-sensitive calcium channels involves tyrosine phosphorylation. Eur. J. Neurosci..

[38] Toescu E.C. (1999). Activity of voltage-operated calcium channels in rat cerebellar granule neurons and neuronal survival. Neuroscience.

[39] D’Mello S.R., Galli C., Ciotti T., Calissano P. (1993). Induction of apoptosis in cerebellar granule neurons by low potassium: Inhibition of death by insulin-like growth factor I and cAMP. Proc. Natl. Acad. Sci. USA.

[40] Chang J.Y., Korolev V.V., Wang J.Z. (1996). Cyclic AMP and pituitary adenylate cyclase-activating polypeptide (PACAP) prevent programmed cell death of cultured cerebellar granule cells. Neurosci. Lett..

[41] Campard P.K., Crochemore C., Rene F., Monnier D., Koch B., Loefer J.P. (1997). PACAP type I receptor activation promotes cerebellar neuron survival through the cAMP/PKA signaling pathway. DNA Cell Biol..

[42] Contestabile A. (2002). Cerebellar granule cells as a model to study mechanisms of neuronal apoptosis or survival in vivo and in vitro. Cerebellum.

[43] Yano S., Tokumitsu H., Soderling T.R. (1998). Calcium promotes cell survival through CaM-kinase activation of the protein-kinase B pathway. Nature.

[44] See V., Boutillier A.R., Bito H., Loefer J.P. (2001). Calcium/calmodulin-dependent protein kinase IV (CaMKIV) inhibits apoptosis induced by potassium deprivation in cerebellar granule neurons. FASEB J..

[45] Gutierrez-Merino C., Marques-da-Silva D., Fortalezas S., Samhan-Arias A.K. (2016). The critical role of lipid rafts nanodomains in the cross-talk between calcium and reactive oxygen and nitrogen species in cerebellar granule neurons apoptosis by extracellular potassium deprivation. AIMS Mol. Sci..

[46] Anderson M.E., Braun A.P., Wu Y., Lu T., Wu Y., Schulman H., Sung R.J. (1998). KN-93, an Inhibitor of Multifunctional Ca^++^/Calmodulin-Dependent Protein Kinase, Decreases Early After Depolarizations in Rabbit Heart. J. Pharm. Exp. Ther..

[47] Samhan-Arias A.K., Martin-Romero F.J., Gutierrez-Merino C. (2004). Kaempferol blocks oxidative stress in cerebellar granule cells and reveals a key role for the plasma membrane NADH oxidase activity in the commitment of apoptosis. Free Radic. Biol. Med..

[48] Martin-Romero F.J., Garcia-Martin E., Gutierrez-Merino C. (2002). Inhibition of the oxidative stress produced by plasma membrane NADH oxidase delays low-potassium induced apoptosis of cerebellar granule cells. J. Neurochem..

[49] Samhan-Arias A.K., Marques-da-Silva D., Yanamala N., Gutierrez-Merino C. (2012). Stimulation and clustering of cytochrome *b*_5_ reductase in caveolin-rich lipid microdomains is an early event in oxidative stress-mediated apoptosis of cerebellar granule neurons. J. Proteomics.

[50] Coultrap S.J., Bayer K.U. (2012). CaMKII regulation in information processing and storage. Trends Neurosci..

[51] Xiao R.P., Cheng H., Lederer W.J., Suzuki T., Lakatta E.G. (1994). Dual regulation of Ca^2+^/calmodulin-dependent kinase II activity by membrane voltage and by calcium influx. Proc. Natl. Acad. Sci. USA.

[52] Tsujikawa H., Song Y., Watanabe M., Masumiya H., Gupte S.A., Ochi R., Okada T. (2008). Cholesterol depletion modulates basal L-type Ca^2+^ current and abolishes its β-adrenergic enhancement in ventricular myocytes. Am. J. Physiol. Heart Circ. Physiol..

[53] Clark N.C., Nagano N., Kuenzi F.M., Jarolimek W., Huber I., Walter D., Wietzorrek G., Boyce S., Kullmann D.M., Striessnig J. (2003). Neurological phenotype and synaptic function in mice lacking the CaV1.3 alpha subunit of neuronal L-type voltage dependent Ca^2+^ channels. Neuroscience.

[54] Koschak A., Obermair G.J., Pivotto F., Sinnegger-Brauns M.J., Striessnig J., Pietrobon D. (2007). Molecular nature of anomalous L-type calcium channels in mouse cerebellar granule cells. J. Neurosci..

[55] Splawski I., Timothy K.W., Sharpe L.M., Decher N., Kumar P., Bloise R., Napolitano C., Schwartz P.J., Joseph R.M., Condorius K. (2004). Ca(V)1.2 calcium channel dysfunction causes a multisystem disorder including arrhythmia and autism. Cell.

[56] Thiel W.H., Chen B., Hund T.J., Koval O.M., Purohit A., Song L.S., Mohler P.J., Anderson M.E. (2008). Proarrhythmic defects in Timothy syndrome require calmodulin kinase II. Circulation.

[57] Day M., Wang Z., Ding J., An X., Ingham C.A., Shering A.F., Wokosin D., Ilijic E., Sun Z., Sampson A.R. (2006). Selective elimination of glutamatergic synapses on striatopallidal neurons in Parkinson disease models. Nat. Neurosci..

[58] Moosmang S., Haider N., Klugbauer N., Adelsberger H., Langwieser N., Müller J., Stiess M., Marais E., Schulla V., Lacinova L. (2005). Role of hippocampal Cav1.2 Ca^2+^ channels in NMDA receptor-independent synaptic plasticity and spatial memory. J. Neurosci..

[59] Ludwig A., Flockerzi V., Hofmann F. (1997). Regional Expression and Cellular Localization of the α_1_ and β Subunit of High Voltage-Activated Calcium Channels in Rat Brain. J. Neurosci..

[60] Abiria S.A., Colbran R.J. (2010). CaMKII associates with CaV1.2 L-type calcium channels via selected β subunits to enhance regulatory phosphorylation. J. Neurosci..

[61] Soltis A.R., Saucerman J.J. (2010). Synergy between CaMKII Substrates and β-Adrenergic Signaling in Regulation of Cardiac Myocyte Ca^2+^ Handling. Biophys. J..

[62] Samhan-Arias A.K., Garcia-Bereguiain M.A., Martin-Romero F.J., Gutierrez-Merino C. (2009). Clustering of plasma membrane-bound cytochrome *b*_5_ reductase within ‘lipid rafts’ microdomains of the neuronal plasma membrane. Mol. Cell. Neurosci..

[63] Fortalezas S., Marques-da-Silva D., Gutierrez-Merino C. (2018). Creatine protects against cytosolic calcium dysregulation, mitochondrial depolarization and increase of reactive oxygen species production in rotenone-induced cell death of cerebellar granule neurons. Neurotox. Res..

