# Supplementary material for: Methyl-β-Cyclodextrin Impairs the Phosphorylation of the β2 Subunit of L-Type Calcium Channels and Cytosolic Calcium Homeostasis in Mature Cerebellar Granule Neurons
Source: Int J Mol Sci. 2018 Nov 20;19(11):3667. doi: 10.3390/ijms19113667 (PMC6275079; doi:10.3390/ijms19113667)
Supplement: Supplementary file 1 [file ijms-19-03667-s001.pdf]

<u>Supplementary Figure</u>. The steady-state $[Ca^{2+}]_i$ of CGNs in MLocke's K25 is largely dependent on calcium entry through LTCCs. The population averages of neuronal somas show that the addition of the specific LTCC blocking agent nifedipine produces by far the largest drop in the 340/380 ratio, i.e., 0.55 ± 0.05. The NMDAr inhibitor MK-801 elicits only a minor decrease in the steady-state 340/380 ratio in the CGN somas of 0.15 ± 0.05, while the addition of the N/P/Q-VGCC blocking agent ω-conotoxin MVIIC does not produce a statistically significant decrease in the steady-state 340/380 ratio, e.g., at most, a weak decrease in the 340/380 ratio of 0.1 (less than twice the average size of error bars).

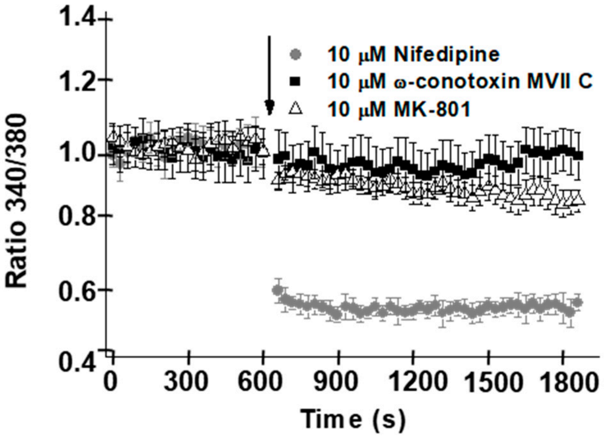

Mature CGNs in culture were loaded with Fura-2, as indicated in the Materials and Methods section, and then changed to MLocke's K25 buffer (37 ºC) and treated with the indicated specific inhibitors of calcium entry systems. The population averages of 340/380 ratio measurements in neuronal somas were measured before and after the addition of specific inhibitors of the major calcium entry systems under our experimental conditions. The kinetic analysis of the 340/380 ratio in the CGN somas was performed before and after the addition of 10 μM nifedipine (solid gray circles), 10 μM ω-conotoxin MVIIC (solid black squares), and 10 μM MK-801 (triangles). The 340/380 ratio data were acquired for cell culture plates treated with each inhibitor, and analysis was carried out after the selection of neuronal somas using the region of interest (ROI) tool of the HCImage software, as indicated in the Materials and Methods section, with exposure times lower than 0.4 s at time intervals of 30 s. The 340/380 ratios shown are the average ± S.E. of experiments carried out with three different preparations of CGNs (n > 300 neuronal somas of fields taken from six plates for each experimental condition).

© 2018 by the authors. Submitted for possible open access publication under the terms and conditions of the Creative Commons Attribution (CC BY) license (http://creativecommons.org/licenses/by/4.0/).
